# S3DB core: a framework for RDF generation and management in bioinformatics infrastructures

**DOI:** 10.1186/1471-2105-11-387

**Published:** 2010-07-20

**Authors:** Jonas S Almeida, Helena F Deus, Wolfgang Maass

**Affiliations:** 1 Department of Bioinformatics and Computational Biology, The University of Texas M D Anderson Cancer Center, 1515 Holcombe Blvd Houston, TX 77030, USA; 2 Institute of Chemical and Biological Technology, Universidade Nova de Lisboa, Oeiras, Portugal; 3 Research Center for Intelligent Media, Furtwangen University, Furtwangen, Germany

## Abstract

**Background:**

Biomedical research is set to greatly benefit from the use of semantic web technologies in the design of computational infrastructure. However, beyond well defined research initiatives, substantial issues of data heterogeneity, source distribution, and privacy currently stand in the way towards the personalization of Medicine.

**Results:**

A computational framework for bioinformatic infrastructure was designed to deal with the heterogeneous data sources and the sensitive mixture of public and private data that characterizes the biomedical domain. This framework consists of a logical model build with semantic web tools, coupled with a Markov process that propagates user operator states. An accompanying open source prototype was developed to meet a series of applications that range from collaborative multi-institution data acquisition efforts to data analysis applications that need to quickly traverse complex data structures. This report describes the two abstractions underlying the S3DB-based infrastructure, logical and numerical, and discusses its generality beyond the immediate confines of existing implementations.

**Conclusions:**

The emergence of the "web as a computer" requires a formal model for the different functionalities involved in reading and writing to it. The S3DB core model proposed was found to address the design criteria of biomedical computational infrastructure, such as those supporting large scale multi-investigator research, clinical trials, and molecular epidemiology.

## Background

The increasing adoption of semantic web technologies and formalisms in biomedical and biomolecular areas is often driven by the need to interoperate between ever more complex data stores and between the applications that process them [1-3]. As the pace of adoption quickens, a distributed infrastructure is emerging that is starting to satisfy the two properties of a von Neumann architecture, also known as "stored-program computer": that it can store both data and the applications. The mingling of data and ready to run applications is particularly tightly woven in web services that rely on padded JSON calls (JSON: Java Script Object Notation), following on proposals for crossdomain JSON calls such as [4], and are now used by major web 2.0 services [5,6]. In those systems, if properly configured [7], there are no syntactic barriers to workflows that pull data and code from different machines and then transfer the results to further use elsewhere.

**Figure 1:**
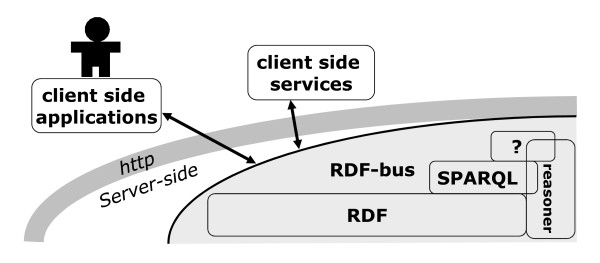
**Web-based infrastructure architecture composed of server side representation and client side presentation + data analysis computational services**. This disposition moves to the client side both the assembly of interfaces as well as the computational intensive data analysis services - such as computational statistics modules. As a consequence, all server side components are standardized and can therefore benefit from cloud computing scaling.

By breaching the same origination restriction of URL calls in the conventional (XML based) AJAX model, JSON-based systems also subvert the client-server equation. Specifically, JSON calls are function calls where the data is passed as the input argument and the function name is specified as a callback parameter in the URL call. This signifies that data and code can be invoked freely from multiple originations and can be made part of arbitrary workflows, as in the node.js project [8]. This outcome, illustrated in Figure 1, was also anticipated by the view of the semantic web as leading to an ecosystem of usages accessing a shared "RDF-bus" [9]. It also suggests an architecture for distributed bioinformatics infrastructures that literally delivers the "web as a computer", that is, a von Neumann machine. Indeed, the work described here can be construed as an attempt to build the minimal set of server-side features that facilitate the integrated management of data and of its analysis in a web infrastructure.

In a strict sense, a von Neumann architecture comes with a von Neumann bottleneck [10] in data access. However, the client side applications described in Figure 1 are hosted in independent machines, that is, in machines with their own memory where data can be cached for ready access by the CPU. Therefore this architecture is more accurately described as a von Neumann hybrid supporting Non-Uniform Memory Access (NUMA). In a nutshell, the distributed computing enabled by web-like architectures have fundamental advantages for scalability that stem from the memory access architecture and the reliance on functional programming (JavaScript) along lines anticipated by John Backus 1977 Turing Lecture, which are now key to data-intensive scientific discovery [11,12].

The use of server-side only as a standardized representation layer for scientific research applications is not original. It is, for example, at the core of cloud computing based systems such as Google Wave [13]. In such systems, the computational intensive data processing components can be deployed as client-side services that regularly consult the representation of the domain they were written to process. In the illustrative Google Wave example those client-side services are designated as "Robots" [14]. What is originally proposed here, and is illustrated with prototype applications, are minimal abstractions that will support the requirements of a distributed bioinformatics infrastructure.

### Design Criteria

The architecture described in this report specifically targets Biomedical applications, which places two requirements on a web-based infrastructure. First, it needs to accommodate the fluidity that is intrinsic to the Biology knowledge domain [15]. Second, individual variability is driving the redesign of biomedical information management systems to allow mixing private and public data in personalized medicine applications [16,17].

The first design criterion of flexible handling of fluid and heterogeneous domains suggests that the Biology domain expert should control the description of the domain that is being experimentally explored. In fact, the results of data analysis often require the redesign of the original data acquisition effort. The redesign happens so patently that, for example, it is now explicitly exploited to speed up target identification and drug discovery through the use of adaptive designs in clinical trials [18]. Redesign is also often triggered in Biomedical research by advances in the analytical methods and can become the major challenge to the use of new technologies, as is currently the case for next generation sequencing [19]. In either case that redefinition of domain has to be achieved without compromising the consistency of the data already acquired. Another consequence of this domain fluidity is the need for co-existence of a myriad of sub-fields and subcommunities which are not necessarily in agreement with each other. This property alone suggests that bioinformatics infrastructure should support bottom-up, collaborative, data acquisition and representation linked to multiple descriptions of the same domain. The motivation for this design criterion is therefore the accommodation of the widest range of data acquisition efforts in the same web-based infrastructure. The resulting resource would be useful as the starting point for the identification of logical models, while not being constrained by them.

The second design criterion, that of fine grained management of access permission, calls for a generic mechanism to describe the relationship between the user and each data element. That description could then be used by the infrastructure to decide what types of access to the data are authorized for each user. A literal reading of this requirement would be to document that relationship for each element, individually, and for each user. This absurd solution would of course increase the size of the data repository several fold. A more scalable alternative is therefore needed which allows for that relationship to be also defined for the description of the domain, and then propagated to its observational instantiation. Accordingly, the identification of a Markov model that propagates user relationships among the S3DB entities is the second key feature of the core model described in this report.

## Methods

The abstractions described in the Results resort to W3C formalisms, are illustrated by an accompanying library and are partially deployed by a web-service:

### Core model entities

The description of the core entities of the S3DB core model was pursued with recourse to the World Wide Web's Consortium (W3C) Resource Description Framework (RDF) [20], including related schema language RDFS [21], and OWL Web Ontology Language [22].

### Propagation of user operators

S3DB operators describe relationships between users and entities of the core model. Any operator predicated on a user as a subject, and on any of the seven S3DB core entities as an object, will be propagated as a Markov process. The propagation model described in the results section was originally coded as a finite state automata (FSA) using MATLAB (Mathworks Inc) and consists of three functions: merge, migrate and propagate. These m-functions were written to be also executable in less sophisticated open source m-code interpreters such as freemat http://freemat.sourceforge.net. The three functions were then also coded in javascript to support web browser based applications such that their inner workings can be explored without need for specialized programming environments. These applications, with links to the m and js source code, are made available at http://s3db-operator.googlecode.com. The Markovian process described by them was used in the prototype web service (also freely provided with open source, see next section) to calculate the independent propagation of each of the three permission operators supported by that particular implementation - *View*, *Edit *and *Use *- for each of the three permission states considered - *none*, *self *and *all*.

### Web service prototype

The identification of the S3DB core model has been pursued for five years as an iterative exercise where tentative new features in the core model were exposed to communities of biomedical and molecular epidemiologists to collect usage feedback [15,23-27]. This feedback typically came with suggestions for desirable behaviors that informed the next round of core model re-design. A regularly updated version of this prototype web service is available for download with open source at the http://s3db.org project web site and also at http://s3db.googlecode.com. The webservice's API is exposed through a REST protocol, S3QL, documented at http://s3db.org/documentation/s3qlsyntax. A javascript library for cross domain JSON requests is also provided at http://s3dbcall.googlecode.com.

## Results

The advantages of a "sloppy", evolvable, data representation distinguishing between domain and instantiation was first argued in [15] using a relational diagram. That argument was expanded and a first draft of the core model was subsequently used to integrate distributed data sources for a Lung Cancer SPORE [24], and to enable the realtime analysis of DNA copy number variation (CNV) in glioblastoma multiforme tumor samples [25], and to support a standards based proteomic repository [26]. A complete model, designated as "s3db core model", needs to merge that draft logical model with the Markovian propagation of user operators used to assign user permissions to S3DB entries.

### Separating Domain from its observational instantiation

**Figure 2:**
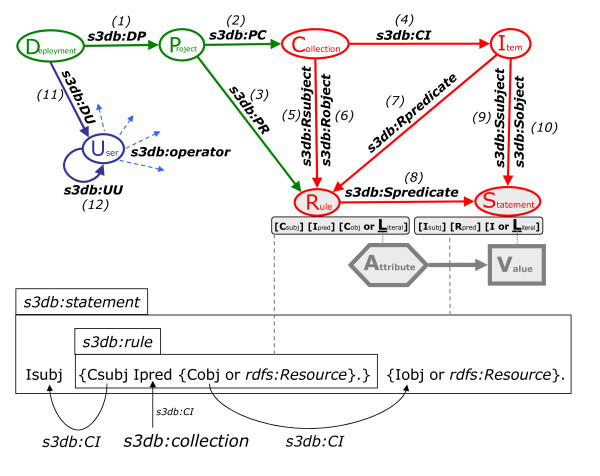
**Two views of the S3db core model**. Top diagram - solid arrows describe relationship between the seven core entities; Dashed arrows (s3db:operator) indicate operators whose states describe the relationship between users and each of the core entities. Bottom box diagram - detail on the key relationship between s3db:rule and s3db:statement using N3 notation. The s3db:rule is a dyadic predicate and it is also, as a whole, the predicate of the s3db:statement. If the object of the s3db:rule triple is not a Collection, then the object of the Statement that rule predicates will be the attribute's literal value. Otherwise the statement's object is the item of the collection indicated as object of the rule. The statement subject is invariably an item from the collection indicated as subject of the predicate rule. See Table 1 for nomenclature and definitions. In the reference prototype (available at s3db.org), the former situation is indeed handled as a literal in a *varchar *database field, as noted in the diagram on top as corresponding to the Attribute/Value model nuclei. However, what that signifies as regards the underlying model is that these two objects can be anything, as are therefore more accurately noted as a rdfs:Resource in the box diagram.

The separation of domain from instantiation is centered on the pattern described in lower half of Figure 2, where the representation of domain as triples is shown to be the predicate of the statements that instantiate that domain with observations. The 7 core entities and 12 logic relationships between them, outlined graphically in Figure 2, are more formally described in Table 1. **In a nutshell, the use of the S3DB model ultimately consists of declaring all the data elements associated with a given observation as being types of S3DB entities**.

**Table 1:** Minimal description of the core 12 relationships and 1 operator between the 7 s3db entities, using notation 3 (N3).

(s3db:deployment s3db:project s3db:collection s3db:item s3db:rule s3db:statement s3db:user) rdfs:subClassOf s3db:entity.
(s3db:DP s3db:PC s3db:PR s3db:CI s3db:CI s3db:Rsubject s3db:Robject s3db:Rpredicate s3db:Ssubject s3db:Sobject s3db:Spredicate) rdfs:subClassOf s3db:relationship.
1. *s3db:DP rdfs:domain s3db:deployment; rdfs:range s3db:project*.
2. *s3db:PC rdfs:domain s3db:project; rdfs:range s3db:collection*.
3. *s3db:PR rdfs:domain s3db:project; rdfs:range s3db:rule*.
4. *s3db:CI rdfs:domain s3db:collection; rdfs:range s3db:item*.
5. *s3db:Rsubject owl:inverseOf rdf:subject; rdfs:domain* *s3db:collection; rdfs:range s3db:rule*.
6. *s3db:Robject owl:inverseOf rdf:object; rdfs:domain* *s3db:collection; rdfs:range s3db:rule*.
7. *s3db:Rpredicate owl:inverseOf rdf:predicate; rdfs:domain* *s3db:item; rdfs:range s3db:rule*.
8. *s3db:Spredicate owl:inverseOf rdf:predicate; rdfs:domain*
*s3db:rule; rdfs:range s3db:statement*.
9. *s3db:Ssubject owl:inverseOf rdf:subject; rdfs:domain s3db:item;* *rdfs:range s3db:statement*.
10. *s3db:Sobject owl:inverseOf rdf:object; rdfs:domain s3db:item;* *rdfs:range s3db:statement*.
11. *s3db:DU rdfs:domain s3db:deployment; rdfs:range s3db:user*.
12. *s3db:UU rdfs:domain s3db:user; rdfs:range s3db:user*.
*s3db:user s3db:operator s3db:entity*.

All relationships except for s3db:operator (last row) are s3db:relationship (first row). The inversion of RDF subject, predicate and object in relations 5-10 may appear capricious at this point but it will simplify the identification of automata for the propagation of s3db:operator states in the next section. Specifically, it will allow the definition of Equation 3 such that the direction of the arrows in Figure 2 is the same as the propagation of s3db:operator states.

#### The core entities

The root entity of each S3DB representation is the Deployment, which is identified by the location of the S3DB service. A Deployment is directly related (first order) with Users and Projects (*s3db:DP *and *s3db:DU *in Figure 2 and Table 1), with the latter providing granularity for sets of Collections and Rules (*s3db:PC *and *s3db:PR*). The Collections are used as subjects and as objects of Rules, which represent the domain one wants to instantiate with observations (*s3db:Rsubject *and *s3db:Robject*). For example the concept that "people live in places" would be represented by a Rule associating the Collection of people with the Collection of locations (see diagram at the bottom of Table 1, and relationships 5-10 in Table 1). The Collections in turn delimit sets of Items (*s3db:CI*). For example, *Mary *could be an Item of the Collection of people. In some cases the object of the rule is best not confined by a Collection and instead can be left as a Literal, as in, for example, the instantiation of the Rule "people have names". Note no cardinality is imposed for any relationship so many-to-many scenarios are allowed. For example, the same Item can be a member, *s3db:CI*, with multiple Collections. On the contrary, we found it useful to restrict the predicates of the Rules, such as *live in *and *have *above, to be Items of Collections (see *s3db:Rpredicate*, #7 in Table 1), as will be clearer after describing the propagation of user operators.

Data submission to a S3DB service corresponds to instantiating those rules with the observations, through the use of Statements. For example, the Statement "Mary lives in Houston" would have a rule like "people live in places" as a predicate associating an Item of the Collection of people (*Mary*) with an Item (*Houston*) of the Collection *place*. One could then continue weaving the description with further assertions by first identifying new components of the domain, for example, by creating a Rule to the effect that "places have addresses" and then asserting those addresses as Items of the Collection of places. It is important to recall that each non-literal element is identified by a Universal Resource Identifier (URI), necessary to make assertions using RDF. In conclusion, the purpose of the S3DB core model is just to provide a template where to aggregate data elements that may already be available, either as their own pre-existing URIs, or, otherwise, by generating those URIs within S3DB.

The illustration of the previous paragraph will now be repeated using the formalism of notation 3 (n3) and the RDF, RDFS and OWL vocabularies (see Methods), with reference to the list of 12 relationships described in Table 1.

We can now return to the example that "Mary lives in Houston" and use the S3DB template to generate the triples to be submitted to the S3DB service. Starting with a deployment hosting an instance of a s3db:project, *P_example*, and using notation 3 (N3),

a) Create Collections of people and places:

*:P_example s3db:PC :C_person*.

*:P_example s3db:PC :C_places*.

b) Insert Mary and Houston as items of the respective collections:

*:C_person s3db:CI :I_Mary*.

*:C_places s3db:CI :I_Houston*.

c) Describe the domain we are about to instantiate, that people live in places, as a

*s3db:rule*:

*:P_example s3db:PR :R_people_in_places*.

*:C_person s3db:Rsubject :R_people_in_places*.

*:C_place s3db:Robject :R_people_in_places*.

*:I_lives_in s3db:Rpredicate :R_people_in_places*.

(lets not worry about what collection of Items *I_lives_in *comes from just yet).

d) Insert the new data:

*:I_Mary :R_people_in_places :Houston*.

This example illustrates a very simple mechanism to store descriptions of the domain and the data that instantiates them in such as way that they can be edited as required by the fluidity of the life sciences domain [15]. The actual identifiers, such as "*:I_Mary*", "*:C_person*" or "*:P_example*" in reality are random or sequential alphanumeric strings such as the unique indexes generated by the S3DB service. Of course *:I_Mary *has a name, which we will use as an example to illustrate how literal values are asserted through instantiation of a Rule, without the need for, say, mediation by a Collection of names:

*@prefix foaf*: <http://xmlns.com/foaf/0.1/>.

*:P_example s3db:PR :R_people_have_ names*.

*:C_person s3db:Rsubject :R_people_have_names*.

*foaf:firstName s3db:Robject :R_people_have_names*.

*:I_has s3db:Rpredicate :R_people_have_names*.

which then allows inserting the corresponding literal information,

*:I_Mary :R_people_have_names "Mary"*.

Note in this last assertion that neither the object of the Rule, *foaf:firstName*, nor the object of the Statement, "Mary", are s3db entities. As noted in Figure 2 and discussed in its legend, when the object of the Rule is not a Collection, the core model allows for any type of content (any other type of *rdfs:Resource*) to be associated to either object in the Rule/Statement instantiation. In the implementation followed by the reference S3DB prototype, these two non-s3db entities are simply stored as literals in a variable length string (*varchar*) database field.

More importantly than the data type of the Rule and Statement objects is that if Mary changes her name that doesn't affect the information about where she lives or that she has a first name. The reverse is also true, if what she has is no longer designated *foaf:firstName*, it could even be replaced by another literal such as "name", that editing does not affect the existing assertion: whatever the new designation is, it is still instantiated in the same Statement, with the same URI, by the same *rdfs:Resource*, in this case the literal "Mary". This is far from being an esoteric scenario. In molecular epidemiology surveillance it is quite common to have, for example, the identity of a microbial isolate, which is an instance of a class, be used as the predicate of the molecular typing methodology. There is of course nothing new here; this modularity is intrinsic to the dyadic predicated nature of the RDF framework. However, what was achieved by tying the submission of new data to the S3DB core model was to restrict the use of RDF such that an explicit distinction of domain and observational instantiation is preserved throughout the process. As will be discussed later, this was achieved purely through the *assertion *[28] of a design pattern, that is, without the computational overhead of description logics and the need of reasoners for subsequent information retrieval.

### Propagation of S3DB operator states

The last relationship in Table 1, *s3db:operator*, is the point of entry for the second component of the core model, the embedded finite state automata (FSA) that propagates the states of any such operator. This component allows the assertion of a generic relationship between a user and a component of the domain, for example, Collections and Rules, and then expect to find it automatically propagated for its instantiation, for example, as Items and Statements (Figure 2). Inversely, S3DB operators can also be used to define exceptions to broader relationships, for example, by using operator states to describe relationships between the User and individual Statements, or Items, without affecting the remainder entries in the same set, that is, for other Statements on the same Rule or Items of the same Collection. This model was identified in very generic terms as to allow for the definition of complex relationships to be described succinctly, without the need to particularize them for each entry. Throughout the different projects where S3DB was used, we have found the critical need for a solution that is balanced between the two extreme scenarios of having a system where all user permissions are indiscriminately set at the point of access, and the extreme alternative of having user group permissions for every entry. Accordingly, the embedded propagation of user relationships was first devised narrowly as a solution for the challenges of mixing public and private data, as well as mixing data instantiating distinct, even contradictory, descriptions of a domain in multiple investigator initiatives. It was only in the later stages of the project that the opportunity for a generic solution of propagating unspecified operator states became apparent.

An s3db operator, *f*, is a discrete variable with a set of *n *ordered states. The elements of the set can exist in two different forms, a upper case or dominant form, *Φ*, and a lower case or recessive form, *ϕ*. For example, the capital form of the i^th ^state of the operator *f*, would be represented as *Φ_i_*, where *i ε[1,..., n]*. Accordingly, the description of such relationship between a user and some *s3db:entity *is defined using the state of the operator, as represented in Equation 1:(1)

The three functions described below, merge, migrate and percolate, are used in the resolution of state propagation between data elements. That description is best followed by testing different scenarios using the accompanying tool at http://s3db-operator.googlecode.com.

#### Merging

As illustrated in Equation 1, for each user, *U*, and for each instance, *E*, of any of the seven types of *s3db:entity*, the nature of the relationship can be described by an arbitrary number of states of the operator *f *, by simply declaring the *{U f E} *triple. However, regardless of such statements having been made between a User and an Entity, the *f *state assigned as predicate in those statements is not necessarily the effective state of that relationship. Other states may also be indirectly asserted to the relationship by directly assigning them for relationships with entities upstream of the target entity. The resolution of what state is effective for the relationship between a given *U *and *E *is resolved by merging all the assigned states, directly or indirectly, as defined in Equation 2. In this equation, *A *is the vector of indexes of assigned dominant (upper case) states,Φ; and *a *is the index vector of assigned recessive (lower case) states, *ϕ*. As for the other definitions, the behaviour and implementation of merge can be verified using the accompanying tool at http://s3db-operator.googlecode.com.(2)

The numeric indexes of the vectors *A *and *a*, are integers between 1 and n. However, because numbers are symbols with no upper and lower case, it is easier to represent the resolution of Equation 2 using the alphabetic indexes instead. The argument for using alphabetic indexes is that their case can distinguish between a dominant and a recessive merged state, therefore allowing *a *and *A *to be represented together as a single vector. Two illustrative examples - for an operator with three states indexed as {'b','c','d'}, merge({'b','c','d'}) = merge('d') = 3 and merge({'b','c','C','D'}) = merge('C') = 2. The case of the merged state, 'd' and 'C' in the example, is of no consequence to the operator itself, which will respond only to its position in the ordered state vector, 2 and 3 respectively. If, in this example, the operator was something like *view_query_results() *and the index of the ordered states were {'noView','theCountOnly','yes'}, the result of the first merging might be returned as 'yes' and of the second as view 'theCountOnly'. However, if further operations are to be made on the merged result then the case of the merged state is important and needs to be retained. It is patently easier to return 'd' and 'B', or even 'yes' and 'THECOUNTONLY' than to have to specify that the merged i = 3 was a recessive outcome, whereas i = 2 was dominant, and as a result the state index 2 > 3 when Equation 2 is used.

#### Migration

The direction of the relationships between S3DB entities (Figure 2, Table 1) was conveniently defined to be the same as the propagation of operator states from domain to its instantiation (note inversion of *rdf:subject*, *rdf:predicate *and *rdf:object *in relationships 5-10, Table 1). This allows the definition of a Boolean transition matrix, Equation 3, that can be applied to any instance of one or more of the seven types of s3db Entity, *E*, ordered using their initials as *[D, P, C, R, I, S, U]*. The numbers between brackets in the transition matrix indicate the logical tests (as numbered in Table 1) that individual instances of the seven types of entities can have between each other (Figure 2).(3)

As described in Equation 4, this simplifies the computation of the transition of states between entities as the external product of the corresponding Boolean square matrix and the vertical vector of states assigned to each entity. For example, if a state of a *s3db:operator *is used to describe a User relation with a certain *s3db:collection*, and this Collection happens to both have Items and to be the Subject of a Rule, then this state will be passed to those Rules and to those Items, using relationships (4) and (5), respectively.(4)

The process by which states are passed from one instance of an entity to another before being merged at the end of each iteration (Equation 4) is designated as state migration and is described in Equation 5. The simplest example is the migration of a singular state - if the state of an instance of a *s3db:entity*, *E*, is described as a singular value, say 'a', then 'a' will be passed on for the relationships verified in Equation 4. However, if the state of the operator, *f*, is described by more than one value, *l>*1, then the additional expressivity in state propagation can be achieved, as described by Equation 5. That generalization consists of specifying that if a state is singular (*l = *1), then it will be passed as is. If, on the other hand, it is plural, then the first state is used as the effective state of the subject entity and only the remaining states are passed on to the entity that is object of the valid relationship, as described in Equation 5. For example, starting with singular migration, if the state of an instance is 'a', and this instance is subject of one or more of the 12 relationships (Table 1), then the object state will merged with the migrated state 'a'. However, if the subject instance has a plural state, say 'abCd', then only 'bCd' will migrate. Note that both dominant and recessive cases are considered in the vector *f *in Equation 5.(5)

One last generalization of the migration process was also found to increase expressivity. The procedure described in Equation 5 was vectorized to allow simultaneous migration of states of multiple s3db operators. This is achieved by defining a second input argument for the *migrate *procedure which identifies how many operators *f_j_*, *j = 1,...,m*, are having their states migrated simultaneously. Since the states of each operator define m-tupples inside the state of *n *states, this is equivalent to identifying the migrating states of *f_j _*as being f_j _=f[i,i+m,i+2 m,i+3 m,...,n*m]. Accordingly, Equation 6 is equal to Equation 5 when m = 1, that is, when only one operator is being considered.(6)

The enhanced expressiveness of the representation of multiple operator states described in Equation 6 is most useful for s3db operators that share the same states. For example, states that identify groups of users could be used as the states of multiple operators such as "view" and "edit", as is the case for the S3DB prototype (see Methods). As can be verified in the tool accompanying this manuscript, the multiple state migration allows for very short descriptions of states that span multiple operators. For example, migrate('a',3) = 'aaa', which allows for a single assignment that spans several operators. This is achieved without affecting the migration of individual statements - for example migrate('abc',3) = 'abc'- while at the same time allowing for a sweeping assignment of migrated states as in migrate('abcb',3) = 'bbb'. Note also in Equation 6 that when a operator state at position *i *is not specified, it is borrowed from the operator immediately to the left, position *i*-1. This implies that the order of the operators can be used to simplify assignments that just span a subset of them, as in migrate('abcbc',3) being 'bcc'. As always, the behavior and implementation of this functionality can be verified using the accompanying tool.

#### Percolate

The third and last function used by the state propagation procedure brings together the merge and migration functions to find the steady state solution of Equation 4. That is, when the migration of states, Equation 6, has progressed to the point where the effective state of the operator, for each and every s3db entity, no longer changes:(7)

In the accompanying web tool this resolution is made available for any Boolean transition matrix. Although for the specific purposes of the S3DB prototype, the transition matrix *T *in Equation 4 is equal to *TS3DB *in Equation 3, there is no reason not to define, and test using the tool, the percolation of s3db operator states more broadly for arbitrary transitions.

## Discussion

The S3DB framework comprises a core model with an embedded Markov process propagating user operator states. The two key properties of the resulting construct are the explicit separation between domain and its experimental instantiation, and the accommodation of a very flexible and fine tuned description of the relationship between the users and its contents. Most features of this framework were put to use in an open source prototype available at s3db.org. They have also been validated with practical applications developing multiple investigator information management infrastructure such as [24]. The potential usages and configurations of the S3DB framework described here are nevertheless much broader and can be described as a set of restrictions placed on RDF representations. Recalling from the Results section, **the use of the S3DB model consists of declaring all the data elements associated with a given observation as being types of S3DB entities**. The interoperability with the resulting construct is ideally delivered as a REST web service protocol, which is not covered in this report. For an illustrative implementation see the prototype's documentation for the query language S3QL, to which SPARQL queries can also be mapped http://sparql.s3db.org.

### Core model

As described in the box diagram at the bottom of Figure 2, the key feature of the core model is the representation of value triple statements (*s3db:statements*) predicated on triple statements that describe the domain instantiated (*s3db:rules*). This design pattern was specifically devised to allow domain experts to incubate the description of their own domain of expertise [15]. As proposed in that report, and verified here, that pattern establishes a specific relationship between the Objects and Subjects between the two triples, which allows the autonomous editing of the domain description and of its value instantiation.

The three core s3db entities peripheral to the nuclear square of Collections, Items, Statements and Rules (in red in Figure 2) create additional management modeling opportunities without which the core model would be little more than a flexible data format. The first of these three entities is the Deployment, which was conceived as a pointer to the URL of the S3DB web service. As all model entities, its usage is not conditioned, nor does it condition, the location of the other entities (Users, Projects and Rules in this case) linked to it. This design seeks to support the distribution of the information management infrastructure. This can be achieved for example, by dereferencing. The Deployment URL address can point to a central registry that resolves it to the actual web service. In that case, the content hosted can be distributed between multiple machines for purposes of, say, load balancing, or in general to enable intermediate tools that may aid in content discovery. Another example is the compartmentalization of content by hosting Collections and Rules in multiple machines, distinct from the hosting Projects (in green in Figure 2). Specifically, because the project URI is resolved by the hosting deployment, the relationship *s3db:DP *(Table 1) can be used to associate deployments with arbitrary Projects, which do not have to be in the same machine that hosts the subject Deployment. Furthermore, by defining URI's for individual entities as URL calls to the Deployment this discussion is extensible to all other relationships in Table 1. This can also be verified by following this link [29] to a S3DB project (click Enter on the key field to login as a public user), with content retrieved from The Cancer Genome Atlas (TCGA). Note the resolved URI's at the lower left corner of the web application.

The last of the four peripheral Entities is the User which is *rdf:subject *of a class of operators with states that were conceived for fine grained definition of relationship between users and the corresponding content. As discussed above for Deployments, Collections and Rules, the URI of a user can also be resolved to any deployment by *s3db:DU *(#11 in Table 1). This implies that authentication and user management are architecturally decoupled. The users have also specified a relationship between themselves, *s3db:UU *(#12 in Table 1), conceived to support a flexible definition of grouping. When two users are linked by *s3db:UU*, the operator states can migrate between them to extend or restrict the relationships with the corresponding content. This enables the use of *s3db:UU *to design flexible user management systems. For example, if one User entity is used as a hub to which multiple UU relationships converge, this is akin to the conventional definition of groups. The reverse would be closer to the conventional definition of roles. A rich variety of groupings can be envisioned between these two conventional extremes. Note that contrary to the operator percolation features discussed in the two previous paragraphs, between-user percolation is presently not supported, and therefore was not tested, by the reference S3DB prototype. Two ongoing projects in particular, http://aguia.googlecode.com and http://cnviewer.googlecode.com, provide S3DB-based, javascript-coded, platforms where those features are being explored, respectively, in the context of clinical trial management and of DNA copy number variation in tumor samples.

### S3DB operators

The idea of s3db operators as a class of functions with states that describe the relationship between a user and the entities of a data model was conceived independently from its specific application to the S3DB core model. Accordingly, this second component of the core model is applicable to any RDF schema provided that the direction of state propagation is defined (or in its absence by the directionality of RDFS/OWL model, as is the case of the S3DB schema). The generic nature of the S3DB user operators and of the Markov process that propagates its states is apparent in Equations 2,4-7. Similarly, although the S3DB prototype provides an illustrative example using three S3DB operators ('view', 'edit' and 'use'), each with the same three ordered states ('yes', 'self', and 'no'), the range of applications is open ended and is not necessarily associated with permission management. For example, an operator could be defined to represent priority in the retrieval of query results, which could differ between users. By using the states of this operator to define the relationship between a user and the model entities one could configure, for that user, that clinical records with a specific outcome would be, for instance, graphically highlighted. Retrieval priorities, or, for that matter, the choice of graphic interface features, could therefore be personalized by associating them to operator states pointed to the appropriate semantic content.

The graphic presentation beyond access control could also include workflow components. For example, it could be used by a quantitatively minded researcher to have statistics tools automatically applied to the construction of a specialized interface. More interestingly, the concept of User could be used more broadly as that of usage. Quite literally, data analysis procedures could be configured as Users. By treating usages and analytical workflows as users, the corresponding procedure would be automatically executed for content with specified semantics. The same line of discussion can also lead to the observation that there is nothing in the definition of an *s3db:operator *that restricts its use to describe the relationship between a user and the entities of the s3db core model. That restriction is described by the construction of the transition matrix which for this core model happens to be the one defined in Equation 3. Therefore, a different core model would just have to identify a different transition matrix of logical tests and the same state propagation mechanism defined in Equations 2,4-7 would be automatically applicable. In summary, the key feature of the user relation propagation component of the S3DB core model is the articulation of the three functions, merge, migrate and percolate, applied to a set of states that can take a dominant or recessive form. That articulation is defined by those equations and is also illustrated by the accompanying browser-based application at http://s3db-operator.googlecode.com.

## Conclusion

The dyadic predicated nature of Resource Description Framework (RDF) has emerged as the shared representation of a variety of semantic web formalisms and technologies. In this report we describe a core model that mediates the generation and management of the RDF triples by and for domain experts. This model abstraction is the result of five years of bioinformatics infrastructure development in biomedical and molecular epidemiological contexts. The underlying approach/hypothesis is that by explicitly distinguishing description of the domain from its instantiation with observational data, one allows domain experts to freely evolve the former without compromising the actuality of the latter. The other, complementary, critical feature of the S3DB core model is the Markov process that propagates the relationship between users and the entities of a core model. This ability to propagate operators from the description of the domain to its instantiation has already found an immediate application in the management of access permissions. Finally, at the very center of the S3DB abstraction is a two tiered modeling pattern where instances of a class describe the relationship between two classes and, in turn, instances of the resulting triple, which are also triples, host the observed values. This modeling pattern underlies the S3DB schema but may be of more general applicability.

## Authors' contributions

JSA identified the S3DB core model and drafted the manuscript. HFD developed the PHP prototype. WM uncovered and analyzed the model's logical patterns. All authors read and approved the final manuscript.
